# Supplementary material for: Tree islands enhance biodiversity and functioning in oil palm landscapes
Source: Nature. 2023 May 24;618(7964):316–21. doi: 10.1038/s41586-023-06086-5 (PMC10247383; doi:10.1038/s41586-023-06086-5)
Supplement: Supplementary file 1 — Supplementary Notes 1–5, Figs. 1–4 and Tables 1–10. [file 41586_2023_6086_MOESM1_ESM.docx]

**Supplementary Notes**

**Supplementary Note 1: Oil palm yield results for a longer time period**

In order to assess how our oil palm yield results might change when using a different time period, we conducted the analysis for the entire duration of the field measurement campaign (November 2016 - October 2018). Per palm and per area oil yield for this period still indicates a beneficial effect of the experimental treatment on the adjacent oil palms:

The overall effects of the experimental treatment on per palm yield (in kg palm^-1^) and per area yield (in kg ha^-1^) were significant in both cases (χ^2^ = 19.61, df = 2, p-value < 0.001 and χ^2^ = 132.71, df =2, p-value < 0.001, respectively). Multi-comparison indicate that **(i)** per palm yield inside the islands was lower than per palm yield adjacent to the tree islands and per palm yield in the conventional oil palm; **(ii)** per area yield inside the tree islands were lower than per area yield adjacent to the tree islands and per area yield in the conventional oil palm. The distance to the tree island edge (i.e. positions 1, 2 and 3) had an effect on the per palm yield of the adjacent oil palms (χ^2^ = 9.36, df = 2, p-value = 0.009). There was no effect of tree planting on the per palm yield of the adjacent oil palms (χ^2^ = 1.54, df = 1, p-value = 0.21). The effect of oil palm thinning on per palm yield of the adjacent palms was marginally significant (χ^2^ =3.42, df = 1, p-value = 0.06).

As a consequence, the net change in oil palm yield within the tree islands (mean: 770.9 ± 1587 kg island^-1^) remains higher than that of conventional oil palm monocultures (mean: -58.4 ± 52 kg plot^-1^) and the Kuskal-Wallis test indicates no significant differences (p = 0.0858). This suggests that our main conclusion, i.e. the absence of oil palm yield loss at the island and landscape scale, is not an artifact of a particular year.

**Supplementary Note 2: Characteristics of the reference oil palms**

The 30 additional reference palms were evenly distributed across the conventional plantation at approximately equal distance to each tree island (Supplementary Fig. 1) and had a neighborhood that is characteristic of conventional oil palm monocultures (six direct neighbors, see Supplementary Fig. 2).

**Supplementary Note 3: Calculation of per-area oil palm yield**

We calculated the per area yield change between tree islands and reference (kg ha^-1^, Eqn. 1) based on a density-dependent oil palm expansion factor (*EF*, referred to as “oil palm density” in the main text). Because of varying palm densities in different tree islands and also around the islands as compared to the conventional plantation, the *EF* are palm-specific (Eqn. 2 and Eqn. 3). Therein, a scaling-up area A_palm_ is assigned to each palm (Eqn. 4). For reference palms, the conventional planting scheme is unaffected by plot-thinning, so a single palm represents one out of 120 planted palms per ha, which leads to A_palm_ equal to the inverse of planting density and one single expansion factor 𝐸𝐹_ref_ equal to 120 (Eqn. 5). For palms inside tree islands, simply using island area divided by the number of palms as *A_palm_* is not appropriate because of the influence of island position and orientation relative to the planting scheme. We therefore applied a more robust approach for estimating A_palm_ by using a virtual search circle with a radius r = 12 m (Supplementary Fig. 2) around each palm.

This radius was chosen as the result of exploratory simulations: According to the planting scheme, the distance from a given palm to its six closest neighbors is 9.8 m, but some buffer should be included to account for inaccuracies during planting and position measurements. However, expanding the radius beyond 17 m would lead to additional neighbor palms being included. To determine the optimal radius for our study, we tested different radii within the range between 9.8 m and 17 m and observed the impact on changes in the number of included palms. Based on the results, we chose a radius of 12 m for the virtual search circles in our study (Supplementary Fig. 3).

Building on the uniform equilateral triangular planting scheme in the conventional plantation, a 12m radius circle is assumed to host seven palms, i.e. a given palm of interest plus six neighbors. Where all seven palms were present, the EF is equal to 𝐸𝐹_ref_. Each palm less within the circle reduces the EF by one seventh (Eqn. 6). A palm with two removed neighbors would for example result in 𝐸𝐹_thinning_ equal to 86.

| ∆𝑌_ha_ = 𝑌_ℎ𝑎_ − 𝑌_ℎ𝑎_ref_ | **(1)** |
| --- | --- |
| 𝑌_ℎ𝑎_ref_ = 𝐸𝐹_ref_ * 𝑌_𝑝___ref_ | **(2)** |
| 𝑌_ℎ𝑎_ = 𝐸𝐹 * 𝑌_𝑝_ | **(3)** |
| 𝐸𝐹_𝑔𝑒𝑛𝑒𝑟𝑎𝑙_ = 𝐴_ℎ𝑎_ / A_palm_ | **(4)** |
| 𝐸𝐹_ref_ = 𝐴_ℎ𝑎_ * 𝑑_𝑝𝑙_ _ℎ𝑎_ = 120 | **(5)** |
| 𝐸𝐹 = 𝐸𝐹_ref_(7− 𝑛_𝑐𝑢𝑡_ / 7) | **(6)** |

∆𝑌_ha_ = per area yield change between tree islands and references (kg ha^-1^)

𝑌_ℎ𝑎_ = per area yield of palms in the tree islands (kg ha^-1^)

𝑌_ℎ𝑎_ref_ = per area yield of reference palms (kg ha^-1^)

𝑌_ℎ𝑎_ = per area yield of palms in the tree islands (kg ha^-1^)

𝑌_𝑝_ = per palm yield of reference palms (kg ha^-1^)

𝑌_𝑝_ref_ = per palm yield of palms in the tree islands (kg ha^-1^)

𝐸𝐹_𝑔𝑒𝑛𝑒𝑟𝑎𝑙_ = general expansion factor

𝐸𝐹 = expansion factor for palms in the tree islands

𝐸𝐹_ref_ = expansion factor for reference palms 𝐴_ℎ𝑎_ = target area (ha)

A_palm_ = scaling-up area of one palm (ha)

𝑑_𝑝𝑙 ℎ𝑎_ = conventional (‘reference’) palm planting density (ha^-1^)

𝑛_𝑐𝑢𝑡_ = number of removed palms i within circle of r = 12 m around palm

**Supplementary Note 4: Cluster analysis on ecosystem functioning indicators**

In order to identity cluster of related ecosystem functioning indicators, we performed a hierarchical cluster analysis on dissimilarities calculated based on the Euclidian distance between indicators. The resulting dendrogram (Supplementary Fig. 5) indicates that tree growth formed a close cluster with litter input. Therefore, tree growth was excluded from the analyses.

**Supplementary Note 5: Results from the Kruskal-Wallis tests**

As an alternative to the linear mixed effect models, we applied Kruskal-Wallis tests on each indicator of biodiversity and ecosystem functioning for comparison between the 52 experimental tree islands and the four conventionally managed oil palm as control plots. Our results (Supplementary Tables 4 and 5) show that a statistically significant increase in tree and bird species richness (χ^2^ = 9.36, p=0.002 and χ^2^ = 5.4 p=0.020) and decreases in the diversity of most abundant seed species (χ^2^ = 4.6802, p = 0.03 for Simpson diversity). Restoration benefits of tree islands were also found for water infiltration (χ^2^ = 4.15; p = 0.04), litter input (χ^2^ = 4.54; p = 0.03), activity of insectivorous bats and birds (χ^2^ = 3.77; p = 0.05) and soil fertility (χ^2^ = 6.15; p = 0.01).

**
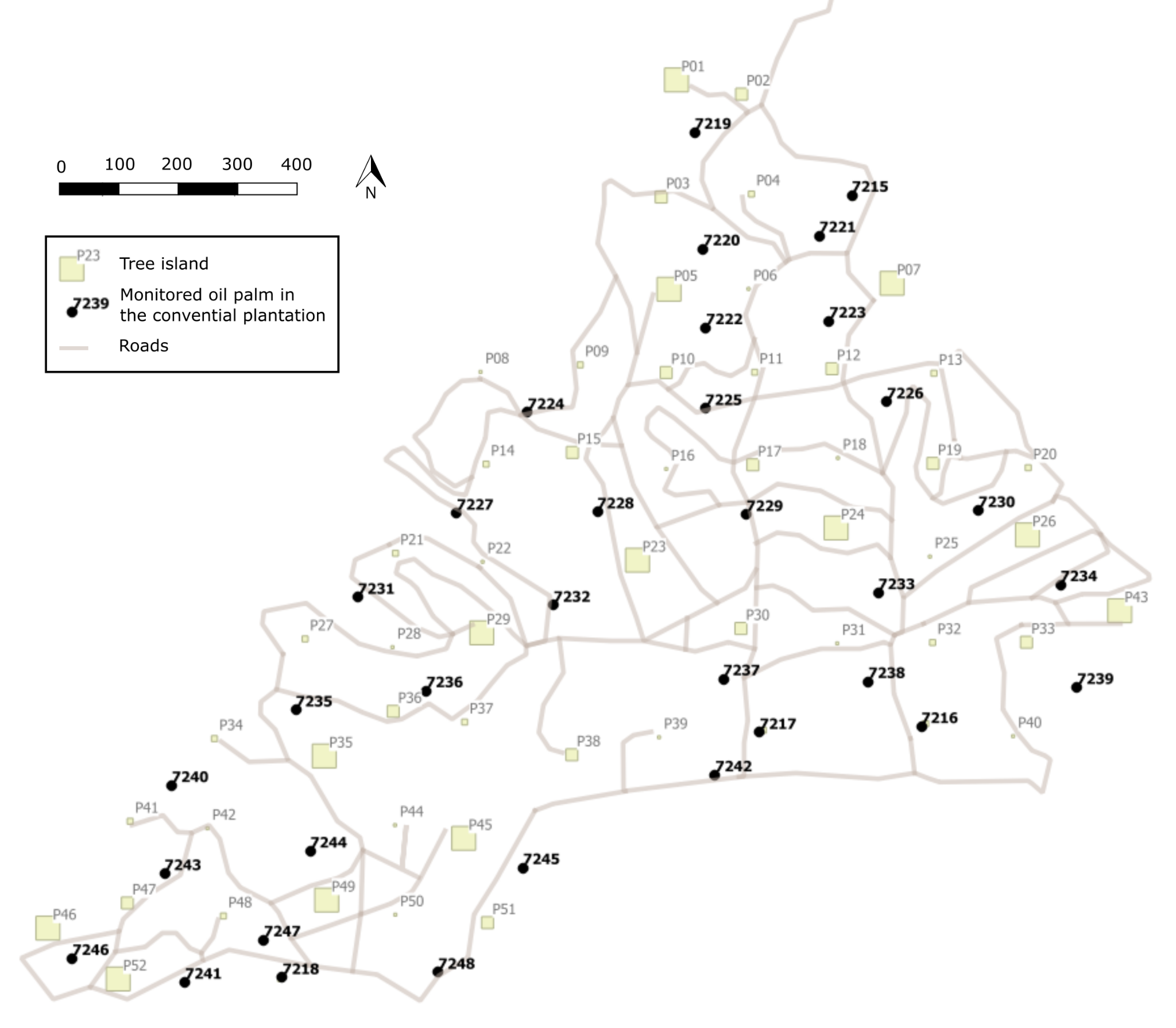
**

**Supplementary Figure 1 |Map of the tree islands and the monitored oil palms in the conventional plantation.** Thirty-four (34) conventionally managed oil palms were monitored, including one palm per control plot (palm number 7215, 7216, 7217 and 7218, corresponding to control plots 52, 53, 54 and 56, respectively) and 30 reference palms at similar distance from each tree island (black dots).

**Supplementary Figures**

**
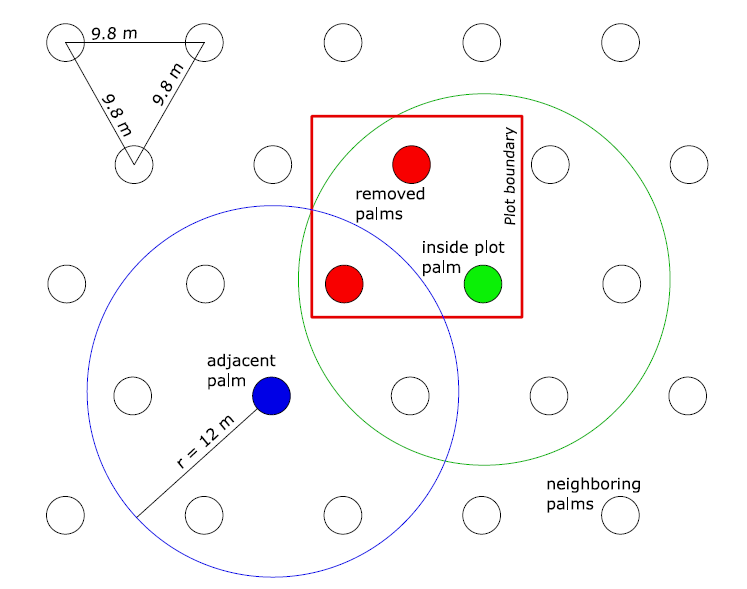
**

**Supplementary Figure 2 | Sketch of the method to estimate the reference area for oil palm density calculations.**

**
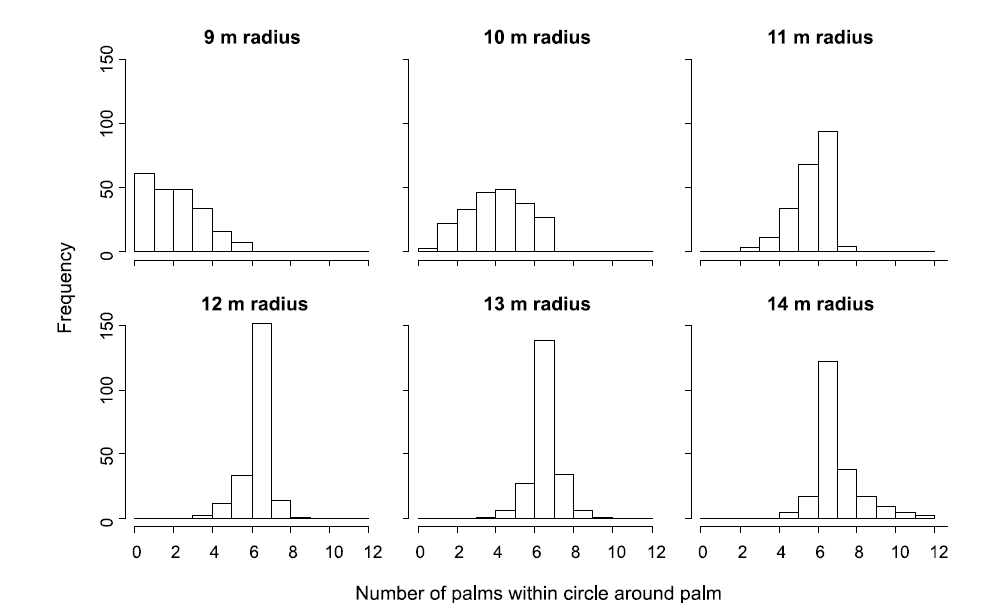
**

**Supplementary Figure 3 | Histograms of palm density within circles around inside plot palms.** The histogram shows the number of palms prior to thinning within circles of different sizes, including the center palm. If planting density (9.8 m x 9.8 m to all six neighboring palms) was as planned, exactly seven palms would be within the circles of all presented histograms. Errors from measurement, planting and modelling, however, cause a deviation from the plan. The optimal radius is r = 12 m because it shows the highest frequency for the intended number of seven palms.


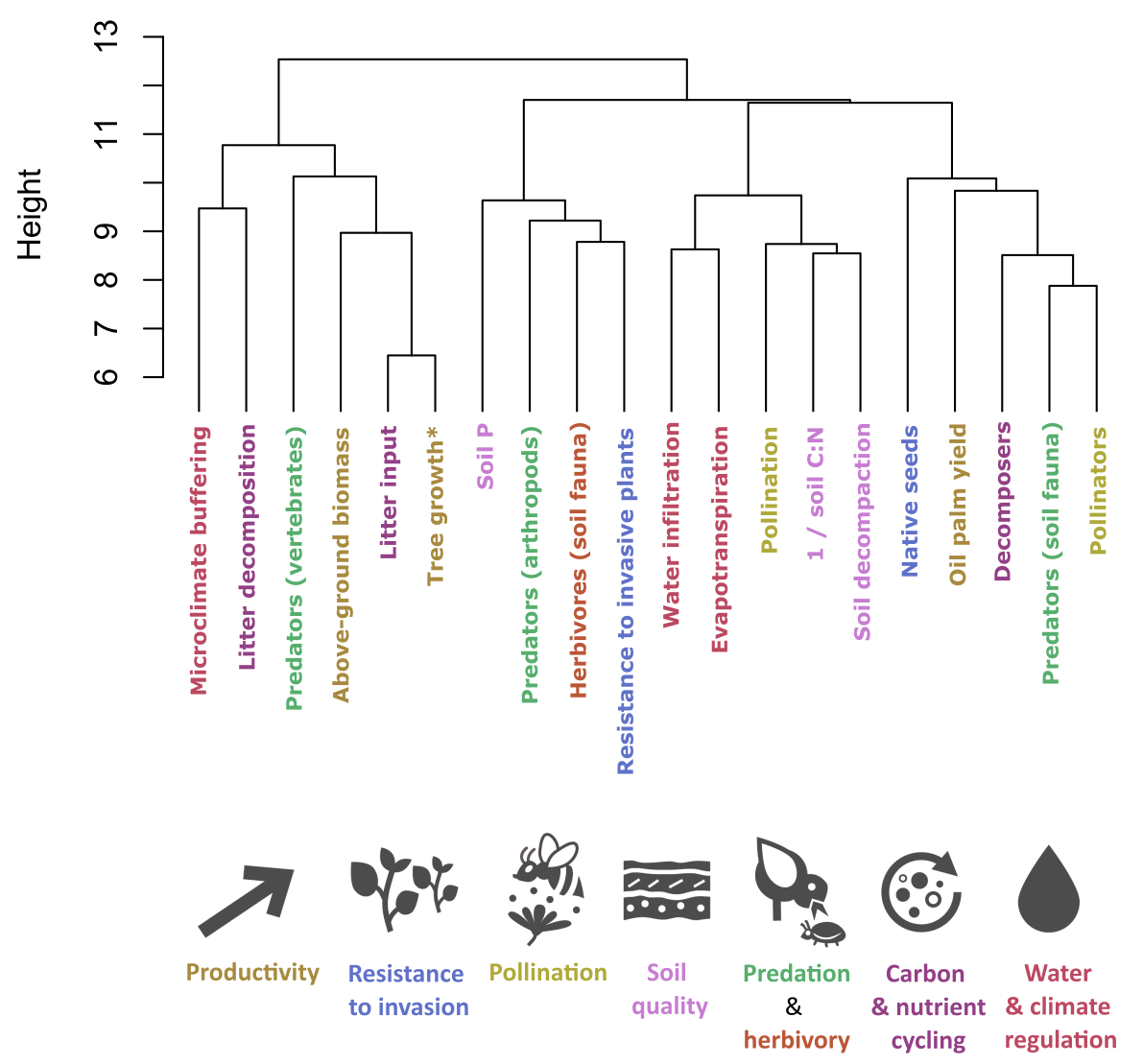


**Supplementary Figure 4 | Cluster analysis of the ecosystem functioning indicators. ***Tree growth was excluded from the analysis because of its close clustering with litter input.

**Supplementary Tables**

**Supplementary Table 1.** List of the biodiversity indicators.

| **Indicator name** | **Method in the field** | **Level** | **Period** | **Link to access raw data** |
| --- | --- | --- | --- | --- |
| Bats | Sound recorders | Plot | March-2017 | <https://ecosound-web.de/ecosound_web/collection/show/36> |
| Birds | Sound recorders | Plot | March-2017 | <https://ecosound-web.de/ecosound_web/collection/show/25> |
| Arthropods | Pan traps | Plot | Oct-16 to Jan-17 | <https://doi.org/10.25625/WUZLJI> |
| Herbs | Understorey vegetation inventory | Sub-plot | March-2018 | <https://doi.org/10.25625/1JX6JK> |
| Pollen | Pollen traps | Plot | Jun-18 to Oct-18 | <https://doi.org/10.25625/JZSMMR> |
| Seeds | Litter traps | Plot | Apr-17 to Mar-18 | https://doi.org/10.25625/TOV012 |
| Trees | Tree inventory | Plot | Apr-18 to Aug-18 | <https://doi.org/10.25625/CKJXTW> |
| Soil fauna | Gradient heat extractor | Sub-plot | Oct-16 to Nov-16 | <https://doi.org/10.25625/9CER2F> |
| Soil bacteria | DNA analysis from soil samples | Sub-plot | 2017 | <https://doi.org/10.25625/C7EDJH> |
| Soil fungi | DNA analysis from soil samples | Sub-plot | January-17 | <https://doi.org/10.25625/K5B41> |

**Supplementary Table 2.** List of the ecosystem functioning indicators. Note that the 20th indicator (“tree growth”) is excluded from the analysis due to close clustering with litter input.

| **Indicator name and description** | **Indicator short name** | **Category** | **Method in the field** | **Level** | **Unit** | **Period** | **Link to access raw data** |
| --- | --- | --- | --- | --- | --- | --- | --- |
| Per island oil palm yield change | Oil palm yield | Productivity | Weighting of fresh fruit bunches (FFB) after harvest | Plot (and adjacent palms) | kg island^-1^ | Nov-17 to Oct-18 | <https://doi.org/10.25625/ZD1EMP> |
| Aboveground biomass of planted trees and oil palms | Aboveground biomass | Productivity | Measurement of height and diameter at breast height of all planted trees and palms inside plot | Plot | t ha^-1^ | January-17 | <https://doi.org/10.25625/IX5QDR> |
| Density of native seeds | Native seeds | Resistance to invasion | Seed traps | Plot | N m^-2^ | Apr-17 to Mar-18 | <https://doi.org/10.25625/TOV012> |
| 100 - Cover of *Clidemia hirta* | Resistance to invasive plants | Resistance to invasion | Estimation ground cover | Sub-plot | % | March-18 | <https://doi.org/10.25625/CKJXTW> |
| Abundance of pollinators | Pollinators | Pollination | Pan traps | Plot | N | Oct-16 to Jan-17 | <https://doi.org/10.25625/WUZLJI> |
| Number of pollinated flowers / total number of flowers | Pollination rate | Pollination | Phytometer plants | Plot | N N^-1^ | Nov.17 | <https://doi.org/10.25625/D7EXMR> |
| Soil available phosphorus content | Soil P | Soil quality | Soil sampling (3 soil cores, 10 cm depth, 4 cm diameter) | Sub-plot | mg g^-1^ | Dec-16 | <https://doi.org/10.25625/OCUDJK> |
| 1 / Soil bulk density | Soil decompaction | Soil quality | Soil sampling (5 soil cores, 100 cm3 ring in 5 cm depth) | Sub-plot | g cm^-3^ | May-18 |  |
| 1 / Soil C:N ratio | 1 / Soil C:N | Soil quality | Soil sampling (3 soil cores, 10 cm depth, 4 cm diameter) | Sub-plot | ratio of mg g^-1^ | Dec-16 | <https://doi.org/10.25625/OCUDJK> |
| Activity of insectivorous bats and birds | Predators (vertebrates) | Predation & herbivory | Sound recording | Plot | sec | Nov-17 and Mar-17 | <https://ecosound-web.uni-goettingen.de/ecosound_web/collection/show/25> |
| Abundance of predators and parasitic understorey arthropods | Predators (arthropods) | Predation & herbivory | Pan traps | Plot | N | Oct-16 to Jan-17 | <https://doi.org/10.25625/WUZLJI> |
| Metabolism of soil decomposers | Predators (soil fauna) | Predation & herbivory | Gradient heat extractor | Sub-plot |  | Oct.17 | <https://doi.org/10.25625/9CER2F> |
| Metabolism of soil herbivores | Herbivores (soil fauna) | Predation & herbivory | Gradient heat extractor | Sub-plot |  | Oct.17 | <https://doi.org/10.25625/9CER2F> |
| Metabolism of soil decomposers | Decomposers | Carbon & nutrient cycling | Gradient heat extractor | Sub-plot | Joul hour^-1^ | Oct.17 | <https://doi.org/10.25625/9CER2F> |
| Litter dry biomass loss after 6 months | Litter decomposition | Carbon & nutrient cycling | Litter bags | Sub-plot | % | Nov-17 to May-18 | <https://doi.org/10.25625/KKE7CU> |
| Leaf litter biomass input | Litter input | Carbon & nutrient cycling | Litter traps (same as seed traps) | Plot | g m^-2^ | Apr-17 to Mar-18 | <https://doi.org/10.25625/7QDKN3> |
| Latent heat flux | Evapotranspiration | Water and climate regulation | Drone-based thermography at mid-day | Plot | Watt m^-2^ | Sept.17 | <https://doi.org/10.25625/H02CO2> |
| Saturated soil hydraulic conductivity | Water infiltration | Water and climate regulation | Dual- head infiltrometer and double-ring infiltrometer | Sub-plot | cm hour^-1^ | March-18 | <https://doi.org/10.25625/O3VCYX> |
| Median daily amplitude of air temperature | Microclimate buffering | Water and climate regulation | Temperature data loggers in plot center | Plot | °C | Nov-17 to Sept-18 | <https://doi.org/10.25625/W0H2GZ> |
| Basal area increment of the planted trees | Tree growth | Productivity | Measurement of basal diameter of all planted trees | Plot | m^2^ ha^-1^ | Jan-17 to Jan-18 | <https://doi.org/10.5061/dryad.2sf02m1> |

| **Variable description** | **Short name** | **Method in the field** | **Level** | **Unit** | **Period** | **Link to access raw data** |
| --- | --- | --- | --- | --- | --- | --- |
| Mean fractal dimension of cross-section polygons, a measure of the geometric complexity of the vegetation structure that is density-dependent | MeanFRAC | Terrestrial laser scanner | Plot |  | Oct-16 | <https://doi.org/10.25625/HOOAJ4> |
| Understorey vegetation cover | Understorey cover | Estimation ground cover | Sub-plot | % | Mar-18 | <https://doi.org/10.25625/1JX6JK> |
| Density of the planted trees | Tree density | Tree inventory | Plot | N ha^-1^ | Jan-18 to Feb-18 | <https://doi.org/10.5061/dryad.2sf02m1> |
| Understorey vegetation complexity index | UCI | Terrestrial laser scanner | Plot |  | Oct-16 | <https://doi.org/10.25625/HOOAJ4> |
| Leaf litter cover | Litter cover | Estimation ground cover | Sub-plot | % | Mar-18 | <https://doi.org/10.25625/1JX6JK> |
| Canopy gap fraction | Gap fraction | Hemispherical photographs | Plot | % | Mar-18 | https://doi.org/10.25625/FSS9ZF |
| Stand structural complexity index | SSCI | Terrestrial laser scanner | Plot |  | Oct-16 | <https://doi.org/10.25625/HOOAJ4> |
| Effective number of layers, a measure of vertical stratification | ENL | Terrestrial laser scanner | Plot |  | Oct-16 | <https://doi.org/10.25625/HOOAJ4> |
| Leaf litter depth | Litter depth | Measurement with a ruler | Sub-plot | cm | Mar-18 | <https://doi.org/10.25625/1JX6JK> |
| Density-dependent oil palm expansion factor | Oil palm density | Drone-based imagery | Plot | N ha^-1^ | Oct-16 | https://doi.org/10.25625/YTKZJO |
| Fraction of the canopy covered by trees | Tree cover | Drone-based structure from motion | Plot | % | Oct-16 | <https://doi.org/10.25625/C06DFF> |
| Fraction of the canopy covered by oil palms | Oil palm cover | Drone-based structure from motion | Plot | % | Oct-16 | <https://doi.org/10.25625/C06DFF> |

**Supplementary Table 3.** List of the vegetation structure variables.

| **Indicator** | **Median** | | | | **Quantile** | | | | **Mean** | | | | **Standard deviation** | |
| --- | --- | --- | --- | --- | --- | --- | --- | --- | --- | --- | --- | --- | --- | --- |
|  | **Control** | **Island** | **Δ** | **%** | **1st control** | **1st island** | **3rd control** | **3rd island** | **Control** | **Island** | **Δ** | **%** | **Control** | **Island** |
| **Species richness** | | | | | | | | | | | | | | |
| Arthropods | 66.5 | 73 | 6.5 | 10 | 63.2 | 61 | 68.5 | 86.8 | 65.2 | 76.2 | 11 | 17 | 5.3 | 18.1 |
| Bats | 2 | 2.5 | 0.5 | 25 | 1.8 | 2 | 2.2 | 3 | 2 | 2.5 | 0.5 | 27 | 0.8 | 0.9 |
| Birds | 0.5 | 3 | 2.5 | 500 | 0 | 1 | 1.2 | 5 | 0.8 | 3.1 | 2.4 | 318 | 1 | 2.2 |
| Herbs | 15 | 13 | -2 | -13 | 14.2 | 9.8 | 15.5 | 16 | 14.8 | 12.9 | -1.9 | -13 | 2.1 | 4.3 |
| Pollen | 8.5 | 7 | -1.5 | -18 | 6.8 | 5 | 10.2 | 10.2 | 8.5 | 7.9 | -0.6 | -7 | 2.4 | 3.9 |
| Seeds | 13 | 17 | 4 | 31 | 13 | 14 | 13.8 | 21 | 13.8 | 17.6 | 3.8 | 28 | 1.5 | 5 |
| Soil bacteria | 13705 | 14305 | 601 | 4 | 13481 | 12829 | 14182 | 15451 | 13959 | 14333 | 375 | 3 | 760 | 2508 |
| Soil fauna | 15 | 17 | 2 | 13 | 14 | 15 | 16.8 | 18 | 15.8 | 16.8 | 1 | 6 | 2.4 | 2.5 |
| Soil fungi | 1581 | 1494 | -87 | -6 | 1433 | 1365 | 1606 | 1643 | 1459 | 1505 | 46.8 | 3 | 277.6 | 218 |
| Trees | 0 | 4.7 | 4.7 |  | 0 | 3 | 0 | 6 | 0 | 4.7 | 4.7 |  | 0 | 2.8 |
| Average multidiversity | 0.5 | 0.6 | 0.1 | 20 | 0.5 | 0.5 | 0.5 | 0.6 | 0.5 | 0.6 | 0.1 | 21 | 0 | 0.1 |
| 20% threshold multidiversity | 7.5 | 9 | 1.5 | 20 | 7 | 8 | 8 | 9 | 7.5 | 8.4 | 0.9 | 12 | 0.6 | 1.1 |
| 50% threshold multidiversity | 2.5 | 4 | 1.5 | 60 | 2 | 3 | 3.2 | 5 | 2.8 | 4.2 | 1.5 | 54 | 1 | 1.4 |
| 80% threshold multidiversity | 0 | 1 | 1 |  | 0 | 0 | 0.2 | 2 | 0.2 | 1.2 | 1 | 392 | 0.5 | 1 |
| **Shannon diversity** | | | | | | | | | | | | | | |
| Arthropods | 21.7 | 23 | 1.3 | 6 | 19.9 | 18.3 | 23.6 | 27.7 | 21.9 | 23.5 | 1.7 | 8 | 2.4 | 6.7 |
| Bats | 2 | 2.4 | 0.4 | 21 | 1.8 | 2 | 2.2 | 3 | 2 | 2.5 | 0.5 | 25 | 0.8 | 0.8 |
| Birds | 0.5 | 2.8 | 2.3 | 465 | 0 | 1 | 1.2 | 4.1 | 0.7 | 2.8 | 2.1 | 289 | 0.9 | 1.9 |
| Herbs | 9.1 | 7.5 | -1.6 | -17 | 8.1 | 4.7 | 10 | 9.2 | 9 | 7.3 | -1.7 | -19 | 2 | 3 |
| Pollen | 1.7 | 1.8 | 0.1 | 6 | 1.6 | 1.4 | 2.2 | 4 | 2 | 2.8 | 0.7 | 35 | 0.8 | 1.9 |
| Seeds | 3.4 | 1.6 | -1.8 | -53 | 2.7 | 1.3 | 4.6 | 2.4 | 3.9 | 2.1 | -1.9 | -48 | 2.1 | 1 |
| Soil bacteria | 3358.7 | 3299 | -59.8 | -2 | 3241.7 | 2977.9 | 3400.6 | 3671.8 | 3283.6 | 3457 | 173.4 | 5 | 238.4 | 958.2 |
| Soil fauna | 5.1 | 6.2 | 1.1 | 22 | 4.9 | 5.2 | 5.4 | 7.2 | 5.1 | 6.2 | 1 | 20 | 0.5 | 1.3 |
| Soil fungi | 162.7 | 179.2 | 16.6 | 10 | 143.3 | 144.5 | 182.1 | 210.6 | 162.7 | 174.2 | 11.5 | 7 | 52.6 | 49.3 |
| Trees | 0 | 3 | 3 |  | 0 | 2.1 | 0 | 4.7 | 0 | 3.4 | 3.4 |  | 0 | 2.2 |
| Average multidiversity | 0.4 | 0.5 | 0.1 | 14 | 0.4 | 0.4 | 0.5 | 0.5 | 0.4 | 0.5 | 0.1 | 16 | 0 | 0.1 |
| 20% threshold multidiversity | 7 | 8 | 1 | 14 | 7 | 6 | 7 | 9 | 7 | 7.5 | 0.5 | 7 | 0 | 1.6 |
| 50% threshold multidiversity | 2.5 | 4 | 1.5 | 60 | 1 | 3 | 4 | 5 | 2.5 | 3.8 | 1.3 | 53 | 1.7 | 1.7 |
| 80% threshold multidiversity | 0.5 | 1 | 0.5 | 100 | 0 | 0 | 1 | 2 | 0.5 | 1.1 | 0.6 | 119 | 0.6 | 1 |
| **Simpson diversity** | | | | | | | | | | | | | | |
| Arthropods | 9.4 | 10.9 | 1.5 | 16 | 8.5 | 7.6 | 10.4 | 13.5 | 9.5 | 10.7 | 1.2 | 13 | 1.7 | 3.9 |
| Bats | 2 | 2.3 | 0.3 | 17 | 1.8 | 2 | 2.2 | 3 | 2 | 2.5 | 0.5 | 24 | 0.8 | 0.8 |
| Birds | 0.5 | 2.7 | 2.2 | 433 | 0 | 1 | 1.2 | 3.9 | 0.7 | 2.6 | 1.9 | 266 | 0.9 | 1.6 |
| Herbs | 7.1 | 4.8 | -2.2 | -32 | 5.7 | 3.5 | 7.9 | 6.5 | 6.5 | 5.2 | -1.3 | -20 | 2.1 | 2.5 |
| Pollen | 1.2 | 1.3 | 0.1 | 7 | 1.2 | 1.2 | 1.4 | 2.5 | 1.4 | 1.9 | 0.5 | 36 | 0.3 | 1.1 |
| Seeds | 2.4 | 1.2 | -1.2 | -49 | 1.7 | 1.1 | 3.2 | 1.8 | 2.6 | 1.5 | -1 | -40 | 1.2 | 0.6 |
| Soil bacteria | 1009.1 | 906 | -103.2 | -10 | 898.7 | 695.6 | 1058.1 | 1185 | 947.8 | 958.6 | 10.8 | 1 | 167.8 | 335.9 |
| Soil fauna | 3.2 | 4.2 | 1 | 33 | 3 | 3.4 | 3.6 | 5 | 3.4 | 4.2 | 0.8 | 24 | 0.6 | 1 |
| Soil fungi | 43.3 | 49.8 | 6.4 | 15 | 28.1 | 35.9 | 60.5 | 64.3 | 45.2 | 49.9 | 4.6 | 10 | 26.8 | 20.6 |
| Trees | 0 | 2.5 | 2.5 |  | 0 | 1.8 | 0 | 3.7 | 0 | 2.8 | 2.8 |  | 0 | 1.9 |
| Average multidiversity | 0.4 | 0.5 | 0.1 | 16 | 0.4 | 0.4 | 0.4 | 0.5 | 0.4 | 0.5 | 0.1 | 18 | 0 | 0.1 |
| 20% threshold multidiversity | 7 | 7 | 0 | 0 | 6.8 | 6.8 | 7 | 8 | 6.8 | 7.3 | 0.5 | 8 | 0.5 | 1.5 |
| 50% threshold multidiversity | 2.5 | 4 | 1.5 | 60 | 1.8 | 2 | 3.5 | 5 | 2.8 | 3.6 | 0.8 | 30 | 1.7 | 1.7 |
| 80% threshold multidiversity | 0.5 | 1 | 0.5 | 100 | 0 | 0 | 1 | 1 | 0.5 | 0.8 | 0.3 | 69 | 0.6 | 0.8 |

**Supplementary Table 4.** Median, first and thrid quantiles, mean and standard deviation values of biodiversity indicators and multidiversity using average multidiversity, and 20, 50, and 80% thresholds multidiversity. Difference (Δ) calculated by island minus control and percentage (%) as difference divided by control multiplied by 100.

| **Indicator** | **Median** | | | | **Quantile** | | | | **Mean** | | | | **Standard deviation** | |
| --- | --- | --- | --- | --- | --- | --- | --- | --- | --- | --- | --- | --- | --- | --- |
|  | **Control** | **Island** | **Δ** | **%** | **1st control** | **1st island** | **3rd control** | **3rd island** | **Control** | **Island** | **Δ** | **%** | **Control** | **Island** |
| Oil palm yield | -46.8 | 297.52 | 344.32 |  | -81.99 | -388.5 | -6.56 | 821.13 | -41.74 | 315 | 356.41 |  | 49.227 | 1370 |
| Aboveground biomass | 48.3 | 44.265 | -3.993 | -8 | 43.089 | 35.645 | 49.336 | 50.798 | 44.17 | 45.7 | 1.543 | 3 | 9.395 | 12.17 |
| Native seeds | 240 | 136.5 | -103.5 | -43 | 65.75 | 62 | 416 | 374.5 | 241.8 | 541 | 299.03 | 124 | 215.19 | 1507.20 |
| Resistance to invasive plants | 82.5 | 70 | -12.5 | -15 | 77.25 | 53.75 | 87.75 | 85 | 82.5 | 68.3 | -14.212 | -17 | 7.141 | 18.55 |
| Pollinators | 21 | 21 | 0 | 0 | 13.75 | 15.5 | 30.75 | 41.75 | 23.5 | 30.5 | 6.962 | 30 | 14.248 | 22.897 |
| Pollination rate | 0.46 | 0.602 | 0.145 | 32 | 0.382 | 0.273 | 0.652 | 0.843 | 0.576 | 0.57 | -0.004 | -1 | 0.448 | 0.382 |
| Soil P | 0.04 | 0.066 | 0.029 | 77 | 0.033 | 0.049 | 0.098 | 0.172 | 0.094 | 0.14 | 0.043 | 46 | 0.119 | 0.152 |
| Soil decompaction | 0.86 | 0.889 | 0.034 | 4 | 0.821 | 0.84 | 0.881 | 0.944 | 0.848 | 0.92 | 0.069 | 8 | 0.099 | 0.117 |
| 1/soil C:N | 0.08 | 0.096 | 0.011 | 14 | 0.084 | 0.09 | 0.087 | 0.1 | 0.086 | 0.1 | 0.01 | 11 | 0.006 | 0.008 |
| Predators (vertebrates) | 36.8 | 241.59 | 204.8 | 556 | 9.3 | 90.078 | 136.67 | 509.07 | 109.2 | 484 | 375.23 | 344 | 168.46 | 644.44 |
| Predators (arthropods) | 23 | 27.5 | 4.5 | 20 | 18.5 | 18.75 | 27 | 38.25 | 22.5 | 31.8 | 9.308 | 41 | 5.26 | 18.56 |
| Predators (soil fauna) | 0.76 | 1.483 | 0.72 | 94 | 0.536 | 0.715 | 0.917 | 2.781 | 0.691 | 2.43 | 1.742 | 252 | 0.333 | 3.241 |
| Herbivores (soil fauna) | 0.04 | 0.053 | 0.017 | 47 | 0.014 | 0.029 | 0.087 | 0.118 | 0.065 | 0.1 | 0.039 | 61 | 0.08 | 0.127 |
| Decomposers | 9.8 | 11.284 | 1.481 | 15 | 7.839 | 6.914 | 12.585 | 17.023 | 10.62 | 12.8 | 2.177 | 20 | 6.98 | 7.305 |
| Litter decomposition | 80 | 70.498 | -9.519 | -12 | 67.51 | 56.472 | 82.723 | 80.934 | 73.48 | 66.4 | -7.063 | -10 | 16.231 | 17.414 |
| Litter input | 4.34 | 10.896 | 6.559 | 151 | 2.32 | 5.512 | 6.102 | 15.805 | 4.085 | 11.7 | 7.66 | 188 | 2.448 | 7.601 |
| Evapotranspiration | 204 | 253.13 | 48.834 | 24 | 134.04 | 180.776 | 246.82 | 332.607 | 176.6 | 275 | 98.231 | 56 | 100.186 | 146.335 |
| Water infiltration | 0 | 0.005 | 0.003 | 174 | 0.001 | 0.002 | 0.003 | 0.007 | 0.002 | 0.01 | 0.004 | 213 | 0.001 | 0.004 |
| Microclimate buffer | 0.15 | 0.147 | 0 | 0 | 0.146 | 0.139 | 0.148 | 0.155 | 0.146 | 0.15 | 0.001 | 1 | 0.003 | 0.014 |
| Average multifunctionality | 0.35 | 0.419 | 0.069 | 20 | 0.345 | 0.386 | 0.365 | 0.464 | 0.36 | 0.42 | 0.062 | 17 | 0.028 | 0.054 |
| 20% threshold multifunctionality | 9 | 12 | 3 | 33 | 8 | 10 | 10.5 | 14 | 9.5 | 11.9 | 2.442 | 26 | 1.915 | 2.024 |
| 50% threshold multifunctionality | 3 | 6 | 3 | 100 | 3 | 4 | 3.5 | 7 | 3.5 | 5.71 | 2.212 | 63 | 1 | 2.003 |
| 80% threshold multifunctionality | 1.5 | 2 | 0.5 | 33 | 1 | 1 | 2 | 3 | 1.5 | 1.85 | 0.346 | 23 | 0.577 | 1.392 |

**Supplementary Table 5.** Median, first and third quantiles, mean and standard deviation values of ecosystem functioning indicators and multifunctionality using average multifunctionality, and 20, 50, and 80% thresholds multifunctionality. Difference (Δ) calculated by island minus control and percentage (%) as difference divided by control multiplied by 100.

**Supplementary Table 6**. Coefficient estimates for piecewise structural equation models explaining biodiversity indicators and multidiversity via tree structural complexity (PC1), tree dominance (-PC2), or alternative mechanisms not included (i.e., direct effects). The influence of planted tree diversity on structural complexity and tree island site on tree dominance were included in all models. df: degree of freedom; Std.Error: standard error; Std.Estimate: Standard estimate.

| **Diversity** | **Response** | **Predictor** | **Estimate** | **Std.Error** | **df** | **Crit.Value** | **p-value** | **Std.Estimate** |
| --- | --- | --- | --- | --- | --- | --- | --- | --- |
| For all piecewise SEM models: | structural complexity (PC1) | planted tree diversity | 0.0979 | 0.0405 | 50 | 2.4199 | **0.0192** | 0.3238 |
|  | tree dominance (-PC2) | tree island size | -0.1111 | 0.0198 | 50 | -5.5971 | **0** | -0.6206 |
| Species richness | Bats | tree island size | -0.3391 | 0.1753 | 49 | -1.9347 | 0.0588 | -0.2654 |
|  |  | planted tree diversity | -0.1902 | 0.2962 | 49 | -0.6424 | 0.5236 | -0.0881 |
|  | Birds | tree island size | 0.1436 | 0.1784 | 49 | 0.805 | 0.4247 | 0.1124 |
|  |  | planted tree diversity | -0.388 | 0.3015 | 49 | -1.2872 | 0.2041 | -0.1797 |
|  | Arthropods | tree island size | 0.1289 | 0.1662 | 47 | 0.7753 | 0.442 | 0.1008 |
|  |  | planted tree diversity | -0.0053 | 0.2337 | 47 | -0.0227 | 0.982 | -0.0025 |
|  |  | structural complexity (PC1) | -3.0666 | 0.7647 | 47 | -4.0102 | **2.00E-04** | -0.4294 |
|  |  | tree dominance (-PC2) | -3.6176 | 0.9356 | 47 | -3.8664 | **3.00E-04** | -0.5066 |
|  | Herbs | tree island size | -0.3524 | 0.1754 | 49 | -2.0091 | 0.0501 | -0.2758 |
|  |  | planted tree diversity | 0.0534 | 0.2964 | 49 | 0.1801 | 0.8578 | 0.0247 |
|  | Pollen | tree island size | 0.0448 | 0.182 | 49 | 0.2461 | 0.8066 | 0.0351 |
|  |  | planted tree diversity | -0.1463 | 0.3076 | 49 | -0.4758 | 0.6364 | -0.0678 |
|  | Seeds | tree island size | -0.3807 | 0.2023 | 47 | -1.8819 | 0.0661 | -0.2979 |
|  |  | planted tree diversity | -0.1029 | 0.2844 | 47 | -0.3618 | 0.7191 | -0.0477 |
|  |  | structural complexity (PC1) | -3.0997 | 0.9307 | 47 | -3.3307 | **0.0017** | -0.4341 |
|  |  | tree dominance (-PC2) | -3.2387 | 1.1387 | 47 | -2.8442 | **0.0066** | -0.4535 |
|  | Trees | tree island size | 0.7668 | 0.1378 | 49 | 5.5657 | **0** | 0.6 |
|  |  | planted tree diversity | 0.5731 | 0.2328 | 49 | 2.4618 | **0.0174** | 0.2654 |
|  | Soil fauna | tree island size | 0.0834 | 0.1813 | 49 | 0.4602 | 0.6474 | 0.0653 |
|  |  | planted tree diversity | -0.2105 | 0.3063 | 49 | -0.687 | 0.4953 | -0.0975 |
|  | Soil bacteria | tree island size | 0.2949 | 0.1776 | 49 | 1.66 | 0.1033 | 0.2307 |
|  |  | planted tree diversity | 0.0264 | 0.3001 | 49 | 0.0879 | 0.9303 | 0.0122 |
|  | Soil fungi | tree island size | -0.0264 | 0.1823 | 49 | -0.1449 | 0.8854 | -0.0207 |
|  |  | planted tree diversity | -0.1039 | 0.308 | 49 | -0.3372 | 0.7374 | -0.0481 |
|  | Average multidiversity | tree island size | -0.0072 | 0.2136 | 48 | -0.0338 | 0.9731 | -0.0057 |
|  |  | planted tree diversity | -0.3721 | 0.2865 | 48 | -1.2988 | 0.2002 | -0.1723 |
|  |  | tree dominance (-PC2) | -3.0786 | 1.2086 | 48 | -2.5473 | **0.0141** | -0.4311 |
|  | 20% threshold multidiversity | tree island size | 0.5821 | 0.1625 | 49 | 3.5834 | **8.00E-04** | 0.4555 |
|  |  | planted tree diversity | 0.0534 | 0.2745 | 49 | 0.1944 | 0.8466 | 0.0247 |
|  | 50% threshold multidiversity | tree island size | 0.0538 | 0.2162 | 48 | 0.2488 | 0.8046 | 0.0421 |
|  |  | planted tree diversity | -0.323 | 0.2901 | 48 | -1.1135 | 0.271 | -0.1496 |
|  |  | tree dominance (-PC2) | -2.6992 | 1.2235 | 48 | -2.2061 | **0.0322** | -0.378 |
|  | 80% threshold multidiversity | tree island size | 0.1213 | 0.181 | 49 | 0.6704 | 0.5058 | 0.0949 |
|  |  | planted tree diversity | -0.1941 | 0.3058 | 49 | -0.6346 | 0.5286 | -0.0899 |
| Shannon diversity | Bats | tree island size | -0.3157 | 0.17 | 48 | -1.8563 | 0.0696 | -0.247 |
|  |  | planted tree diversity | -0.4204 | 0.3018 | 48 | -1.3929 | 0.1701 | -0.1947 |
|  |  | structural complexity (PC1) | 2.0623 | 1.0044 | 48 | 2.0533 | **0.0455** | 0.2888 |
|  | Birds | tree island size | 0.1718 | 0.1781 | 49 | 0.9645 | 0.3396 | 0.1344 |
|  |  | planted tree diversity | -0.3726 | 0.301 | 49 | -1.238 | 0.2216 | -0.1726 |
|  | Arthropods | tree island size | 0.3147 | 0.1645 | 49 | 1.9132 | 0.0616 | 0.2463 |
|  |  | planted tree diversity | -0.7704 | 0.278 | 49 | -2.7714 | **0.0079** | -0.3568 |
|  | Herbs | tree island size | -0.1891 | 0.1708 | 48 | -1.107 | 0.2738 | -0.148 |
|  |  | planted tree diversity | 0.0565 | 0.3032 | 48 | 0.1864 | 0.8529 | 0.0262 |
|  |  | structural complexity (PC1) | 2.4173 | 1.009 | 48 | 2.3958 | **0.0205** | 0.3385 |
|  | Pollen | tree island size | 0.1894 | 0.1805 | 49 | 1.0496 | 0.2991 | 0.1482 |
|  |  | planted tree diversity | -0.0658 | 0.3049 | 49 | -0.2159 | 0.8299 | -0.0305 |
|  | Seeds | tree island size | 0.2837 | 0.1763 | 49 | 1.6097 | 0.1139 | 0.222 |
|  |  | planted tree diversity | 0.2928 | 0.2978 | 49 | 0.9831 | 0.3304 | 0.1356 |
|  | Trees | tree island size | 0.5949 | 0.1535 | 49 | 3.8748 | **3.00E-04** | 0.4655 |
|  |  | planted tree diversity | 0.5958 | 0.2594 | 49 | 2.2968 | **0.0259** | 0.2759 |
|  | Soil fauna | tree island size | 0.3581 | 0.1634 | 49 | 2.1915 | **0.0332** | 0.2802 |
|  |  | planted tree diversity | -0.7494 | 0.2761 | 49 | -2.7146 | **0.0091** | -0.3471 |
|  | Soil bacteria | tree island size | 0.26 | 0.1762 | 49 | 1.4757 | 0.1464 | 0.2035 |
|  |  | planted tree diversity | -0.3545 | 0.2977 | 49 | -1.1906 | 0.2395 | -0.1642 |
|  | Soil fungi | tree island size | -0.0236 | 0.1808 | 49 | -0.1306 | 0.8966 | -0.0185 |
|  |  | planted tree diversity | -0.297 | 0.3055 | 49 | -0.9723 | 0.3357 | -0.1376 |
|  | Average multidiversity | tree island size | 0.4 | 0.1702 | 49 | 2.351 | **0.0228** | 0.313 |
|  |  | planted tree diversity | -0.3941 | 0.2875 | 49 | -1.3709 | 0.1767 | -0.1825 |
|  | 20% threshold multidiversity | tree island size | 0.5446 | 0.1622 | 49 | 3.3587 | **0.0015** | 0.4262 |
|  |  | planted tree diversity | -0.3704 | 0.274 | 49 | -1.3517 | 0.1827 | -0.1715 |
|  | 50% threshold multidiversity | tree island size | -0.1308 | 0.2244 | 48 | -0.5831 | 0.5626 | -0.1024 |
|  |  | planted tree diversity | -0.376 | 0.3011 | 48 | -1.249 | 0.2177 | -0.1742 |
|  |  | tree dominance (-PC2) | -2.568 | 1.2699 | 48 | -2.0221 | **0.0488** | -0.3596 |
|  | 80% threshold multidiversity | tree island size | 0.1375 | 0.1803 | 49 | 0.7623 | 0.4496 | 0.1076 |
|  |  | planted tree diversity | -0.2439 | 0.3047 | 49 | -0.8005 | 0.4273 | -0.113 |
| Simpson diversity | Bats | tree island size | -0.3311 | 0.1689 | 48 | -1.9606 | 0.0557 | -0.2591 |
|  |  | planted tree diversity | -0.4479 | 0.2998 | 48 | -1.4941 | 0.1417 | -0.2075 |
|  |  | structural complexity (PC1) | 2.0846 | 0.9976 | 48 | 2.0895 | 0.042 | 0.2919 |
|  | Birds | tree island size | 0.1969 | 0.1776 | 49 | 1.1083 | 0.2731 | 0.154 |
|  |  | planted tree diversity | -0.3719 | 0.3001 | 49 | -1.2392 | 0.2212 | -0.1722 |
|  | Arthropods | tree island size | 0.2297 | 0.1627 | 49 | 1.4122 | 0.1642 | 0.1798 |
|  |  | planted tree diversity | -0.9003 | 0.2748 | 49 | -3.2756 | **0.0019** | -0.4169 |
|  | Herbs | tree island size | -0.0755 | 0.1731 | 48 | -0.4364 | 0.6645 | -0.0591 |
|  |  | planted tree diversity | 0.1973 | 0.3072 | 48 | 0.6422 | 0.5238 | 0.0914 |
|  |  | structural complexity (PC1) | 2.2238 | 1.0224 | 48 | 2.1752 | **0.0346** | 0.3114 |
|  | Pollen | tree island size | 0.2469 | 0.1789 | 49 | 1.3807 | 0.1736 | 0.1932 |
|  |  | planted tree diversity | -0.1144 | 0.3022 | 49 | -0.3787 | 0.7065 | -0.053 |
|  | Seeds | tree island size | 0.2678 | 0.1762 | 49 | 1.5204 | 0.1348 | 0.2096 |
|  |  | planted tree diversity | 0.3408 | 0.2977 | 49 | 1.1449 | 0.2578 | 0.1578 |
|  | Trees | tree island size | 0.2008 | 0.201 | 48 | 0.9986 | 0.323 | 0.1571 |
|  |  | planted tree diversity | 0.4378 | 0.2697 | 48 | 1.623 | 0.1111 | 0.2027 |
|  |  | tree dominance (-PC2) | -2.5177 | 1.1377 | 48 | -2.213 | **0.0317** | -0.3526 |
|  | Soil fauna | tree island size | 0.3578 | 0.1642 | 49 | 2.1785 | **0.0342** | 0.28 |
|  |  | planted tree diversity | -0.7232 | 0.2775 | 49 | -2.606 | 0.0121 | -0.3349 |
|  | Soil bacteria | tree island size | 0.1446 | 0.1721 | 49 | 0.84 | 0.405 | 0.1131 |
|  |  | planted tree diversity | -0.6766 | 0.2908 | 49 | -2.3265 | **0.0242** | -0.3134 |
|  | Soil fungi | tree island size | -0.0169 | 0.1814 | 49 | -0.0934 | 0.926 | -0.0133 |
|  |  | planted tree diversity | -0.2414 | 0.3065 | 49 | -0.7877 | 0.4347 | -0.1118 |
|  | Average multidiversity | tree island size | 0.3345 | 0.17 | 49 | 1.968 | 0.0547 | 0.2618 |
|  |  | planted tree diversity | -0.5484 | 0.2872 | 49 | -1.9093 | 0.0621 | -0.254 |
|  | 20% threshold multidiversity | tree island size | 0.5152 | 0.1618 | 49 | 3.1845 | **0.0025** | 0.4031 |
|  |  | planted tree diversity | -0.4935 | 0.2733 | 49 | -1.8054 | 0.0772 | -0.2285 |
|  | 50% threshold multidiversity | tree island size | -0.0879 | 0.2224 | 48 | -0.3954 | 0.6943 | -0.0688 |
|  |  | planted tree diversity | -0.3511 | 0.2984 | 48 | -1.1766 | 0.2452 | -0.1626 |
|  |  | tree dominance (-PC2) | -2.6772 | 1.2587 | 48 | -2.1269 | **0.0386** | -0.3749 |
|  | 80% threshold multidiversity | tree island size | 0.1611 | 0.1787 | 49 | 0.9016 | 0.3717 | 0.126 |
|  |  | planted tree diversity | -0.3508 | 0.3019 | 49 | -1.1621 | 0.2508 | -0.1625 |

**Supplementary Table 7.** Coefficient estimates for piecewise structural equation models explaining ecosystem functioning indicators and multifunctionality via tree structural complexity (PC1), tree dominance (-PC2), or alternative mechanisms not included (i.e., direct effects). The influence of planted tree diversity on structural complexity and tree island site on tree dominance were included in all models. df: degree of freedom; Std.Error: standard error; Std.Estimate: Standard estimate.

| **Response** | **Predictor** | **Estimate** | **Std.Error** | **df** | **Crit.Value** | **p-value** | **Std.Estimate** |
| --- | --- | --- | --- | --- | --- | --- | --- |
| Included for all piecewise SEM models: | | | | | | | |
| structural complexity (PC1) | planted tree diversity | 0.0979 | 0.0405 | 50 | 2.4199 | **0.0192** | 0.3238 |
| tree dominance (-PC2) | tree island size | -0.1111 | 0.0198 | 50 | -5.5971 | **0** | -0.6206 |
| Individual piecewise SEM models: | | | | | | | |
| Oil palm yield | tree island size | 0.2117 | 0.1732 | 48 | 1.2223 | 0.2276 | 0.1656 |
|  | planted tree diversity | 0.4212 | 0.3074 | 48 | 1.3703 | 0.177 | 0.1951 |
|  | structural complexity (PC1) | -2.1697 | 1.0228 | 48 | -2.1213 | **0.0391** | -0.3038 |
| Aboveground biomass | tree island size | -0.4046 | 0.1269 | 48 | -3.1887 | **0.0025** | -0.3166 |
|  | planted tree diversity | 0.0079 | 0.2252 | 48 | 0.0352 | 0.9721 | 0.0037 |
|  | structural complexity (PC1) | 4.4489 | 0.7494 | 48 | 5.9363 | **0** | 0.623 |
| Native seeds | tree island size | 0.1325 | 0.1654 | 48 | 0.8009 | 0.4272 | 0.1037 |
|  | planted tree diversity | 0.3908 | 0.2936 | 48 | 1.3312 | 0.1894 | 0.181 |
|  | structural complexity (PC1) | -3.2419 | 0.977 | 48 | -3.3184 | **0.0017** | -0.454 |
| Resistance to invasive plants | tree island size | 0.2925 | 0.1732 | 49 | 1.6885 | 0.0977 | 0.2289 |
|  | planted tree diversity | 0.4698 | 0.2927 | 49 | 1.6053 | 0.1148 | 0.2176 |
| Pollinators | tree island size | 0.2595 | 0.1343 | 48 | 1.933 | 0.0592 | 0.2031 |
|  | planted tree diversity | -0.0212 | 0.2383 | 48 | -0.0888 | 0.9296 | -0.0098 |
|  | structural complexity (PC1) | -4.537 | 0.793 | 48 | -5.7214 | **0** | -0.6353 |
| Pollination rate | tree island size | -0.0621 | 0.1822 | 49 | -0.3409 | 0.7347 | -0.0486 |
|  | planted tree diversity | -0.0921 | 0.3078 | 49 | -0.2993 | 0.766 | -0.0427 |
| Soil P | tree island size | -0.0787 | 0.1822 | 49 | -0.4319 | 0.6677 | -0.0616 |
|  | planted tree diversity | 0.0013 | 0.3079 | 49 | 0.0042 | 0.9967 | 6.00E-04 |
| Soil decompaction | tree island size | 0.2057 | 0.1789 | 49 | 1.1496 | 0.2559 | 0.161 |
|  | planted tree diversity | -0.2489 | 0.3024 | 49 | -0.8231 | 0.4145 | -0.1153 |
| 1/soil C:N | tree island size | 0.0346 | 0.1823 | 49 | 0.1899 | 0.8502 | 0.0271 |
|  | planted tree diversity | -0.0968 | 0.308 | 49 | -0.3142 | 0.7547 | -0.0448 |
| Predators (vertebrates) | tree island size | 0.1304 | 0.1779 | 49 | 0.7333 | 0.4669 | 0.1021 |
|  | planted tree diversity | -0.4334 | 0.3005 | 49 | -1.4421 | 0.1556 | -0.2007 |
| Predators (arthropods) | tree island size | 0.3396 | 0.1647 | 48 | 2.0623 | **0.0446** | 0.2657 |
|  | planted tree diversity | 0.0021 | 0.2923 | 48 | 0.0073 | 0.9942 | 0.001 |
|  | structural complexity (PC1) | -2.5035 | 0.9726 | 48 | -2.5739 | **0.0132** | -0.3506 |
| Predators (soil fauna) | tree island size | 0.1675 | 0.1799 | 49 | 0.9308 | 0.3565 | 0.131 |
|  | planted tree diversity | -0.2314 | 0.304 | 49 | -0.7612 | 0.4502 | -0.1072 |
| Herbivores (soil fauna) | tree island size | 0.4146 | 0.1655 | 48 | 2.505 | **0.0157** | 0.3245 |
|  | planted tree diversity | 0.3107 | 0.2938 | 48 | 1.0574 | 0.2956 | 0.1439 |
|  | structural complexity (PC1) | -2.0945 | 0.9777 | 48 | -2.1424 | **0.0373** | -0.2933 |
| Decomposers | tree island size | 0.4885 | 0.1686 | 49 | 2.8966 | **0.0056** | 0.3823 |
|  | planted tree diversity | 0.0505 | 0.2849 | 49 | 0.1773 | 0.86 | 0.0234 |
| Litter decomposition | tree island size | 0.1231 | 0.1812 | 49 | 0.6792 | 0.5002 | 0.0963 |
|  | planted tree diversity | -0.1558 | 0.3062 | 49 | -0.5086 | 0.6133 | -0.0721 |
| Litter input | tree island size | -0.1786 | 0.1905 | 47 | -0.9379 | 0.3531 | -0.1398 |
|  | planted tree diversity | -0.0714 | 0.2678 | 47 | -0.2665 | 0.791 | -0.0331 |
|  | structural complexity (PC1) | 3.2039 | 0.8763 | 47 | 3.656 | **6.00E-04** | 0.4486 |
|  | tree dominance (-PC2) | -3.4931 | 1.0722 | 47 | -3.2578 | **0.0021** | -0.4891 |
| Evapotranspiration | tree island size | 0.0867 | 0.1808 | 49 | 0.4797 | 0.6336 | 0.0679 |
|  | planted tree diversity | -0.2618 | 0.3055 | 49 | -0.8569 | 0.3957 | -0.1212 |
| Water infiltration | tree island size | 0.2242 | 0.1797 | 49 | 1.2474 | 0.2182 | 0.1754 |
|  | planted tree diversity | 0.042 | 0.3036 | 49 | 0.1382 | 0.8906 | 0.0194 |
| Microclimate buffer | tree island size | -0.124 | 0.1651 | 48 | -0.751 | 0.4563 | -0.097 |
|  | planted tree diversity | -0.4245 | 0.293 | 48 | -1.4487 | 0.1539 | -0.1966 |
|  | structural complexity (PC1) | 3.2766 | 0.975 | 48 | 3.3607 | **0.0015** | 0.4588 |
| Average multifunctionality | tree island size | 0.2149 | 0.1917 | 47 | 1.121 | 0.268 | 0.1681 |
|  | planted tree diversity | -0.0443 | 0.2695 | 47 | -0.1644 | 0.8701 | -0.0205 |
|  | structural complexity (PC1) | -2.1738 | 0.8819 | 47 | -2.4649 | **0.0174** | -0.3044 |
|  | tree dominance (-PC2) | -2.7837 | 1.079 | 47 | -2.5798 | **0.0131** | -0.3898 |
| 20% threshold multifunctionality | tree island size | 0.2353 | 0.1685 | 48 | 1.3961 | 0.1691 | 0.1841 |
|  | planted tree diversity | -0.0026 | 0.2991 | 48 | -0.0086 | 0.9932 | -0.0012 |
|  | structural complexity (PC1) | -2.5552 | 0.9953 | 48 | -2.5672 | **0.0134** | -0.3578 |
| 50% threshold multifunctionality | tree island size | 0.3171 | 0.1753 | 49 | 1.8093 | 0.0765 | 0.2482 |
|  | planted tree diversity | -0.278 | 0.2962 | 49 | -0.9385 | 0.3526 | -0.1287 |
| 80% threshold multifunctionality | tree island size | 0.3145 | 0.1769 | 49 | 1.7771 | 0.0818 | 0.2461 |
|  | planted tree diversity | -0.0041 | 0.299 | 49 | -0.0137 | 0.9891 | -0.0019 |

**Supplementary Table 8.** Comparison of biodiversity indicators and multidiversity (average multidiversity, and 20, 50, and 80% thresholds multidiversity) between conventional oil palm monocultures (control plots, n=4) and tree islands (experimental plots, n=52) using the Kruskal-Wallis test.

| **Indicator** | **Species richness** | | **Shannon diversity** | | **Simpson diversity** | |
| --- | --- | --- | --- | --- | --- | --- |
|  | **chi-squared** | **p-value** | **chi-squared** | **p-value** | **chi-squared** | **p-value** |
| Arthropods | 1,3497 | 0,2453 | 0,2925 | 0,5886 | 0,3279 | 0,5669 |
| Bats | 1,4053 | 0,2358 | 0,9804 | 0,3221 | 0,9804 | 0,3221 |
| Birds | 4,9902 | **0,0255** | 5,4477 | **0,0196** | 5,4472 | **0,0196** |
| Herbs | 0,8562 | 0,3548 | 1,7014 | 0,1921 | 1,2399 | 0,2655 |
| Pollen | 0,233 | 0,6293 | 0,0425 | 0,8368 | 0,1427 | 0,7056 |
| Seeds | 2,495 | 0,1142 | 5,247 | **0,022** | 4,6802 | **0,0305** |
| Trees | 9,3609 | **0,0022** | 9,3606 | **0,0022** | 9,3606 | **0,0022** |
| Soil fauna | 1,021 | 0,3123 | 2,9514 | 0,0858 | 2,7368 | 0,0981 |
| Soil bacteria | 0,2925 | 0,5886 | 0,001 | 0,9746 | 0,1711 | 0,6792 |
| Soil fungi | 0,001 | 0,9746 | 0,1457 | 0,7026 | 0,1012 | 0,7504 |
| Average multidiversity | 9,1346 | **0,0025** | 5,1022 | **0,0239** | 4,8188 | **0,0282** |
| 20% multidiversity | 3,5819 | 0,0584 | 0,7178 | 0,3969 | 1,2005 | 0,2732 |
| 50% multidiversity | 4,5306 | **0,0333** | 1,8427 | 0,1746 | 0,8472 | 0,3573 |
| 80% multidiversity | 3,9224 | **0,0476** | 1,2154 | 0,2703 | 0,571 | 0,4499 |

**Supplementary Table 9.** Comparison of ecosystem functioning indicators and multifunctionality (using average multifunctionality, and 20, 50, and 80% thresholds multifunctionality) between conventional oil palm monocultures (control plots, n=4) and tree islands (experimental plots, n=52) using the Kruskal-Wallis test.

| **Indicator** | **chi-squared** | **p-value** |
| --- | --- | --- |
| Oil palm yield | 0,4899 | 0,484 |
| Microclimate buffering | 0,0041 | 0,9492 |
| Water infiltration | 4,1457 | **0,0417** |
| Evapotranspiration | 1,5395 | 0,2147 |
| Litter input | 4,5435 | **0,033** |
| Litter decomposition | 0,4451 | 0,5047 |
| Decomposers | 0,3654 | 0,5455 |
| Herbivores | 0,4679 | 0,4939 |
| Predators (soil fauna) | 3,0617 | 0,0802 |
| Predators (arthropods) | 0,8832 | 0,3473 |
| Predators (vertebrates) | 3,7662 | 0,0523 |
| 1 / soil C:N | 6,1579 | **0,0131** |
| Soil decompaction | 1,2424 | 0,265 |
| Soil P | 1,9595 | 0,1616 |
| Pollination rate | 0,004 | 0,9493 |
| Pollinators | 0,276 | 0,5994 |
| Resistance to invasive plants | 2,1516 | 0,1424 |
| Native seeds | 0,004 | 0,9493 |
| Aboveground biomass | 0,0648 | 0,7991 |
| Average multifunctionality | 4,9595 | **0,0259** |
| 20% multifunctionality | 3,7324 | **0,0534** |
| 50% multifunctionality | 7,3679 | **0,0066** |
| 80% multifunctionality | 4,3363 | **0,0373** |

**Supplementary Table 10.** List of understorey arthropod groups and associated guild.

| **Group** | | **Guild** |
| --- | --- | --- |
| Araneae | - Amaurobiidae, Araneidae, Lycosidae, Oxyiopidae, Salticidae,  Thomisidae, Uloboridae | Predators |
| Hymenoptera | - Aphelinidae, Braconidae, Ceraphronidae, Chalcididae, Diapriidae,  Dryinidae, Encyrtidae, Eucoilidae, Eulophidae, Evaniidae, Figitidae,  Ichneumonidae, Mymaridae, Platygastridae, Pteromalidae,  Scelionidae, Tenthredinidae, Trichogrammatidae (*Trichogramma*) | Parasitica |
| Hymenoptera | - Apidae (*Amegilla zonata*), Colletidae, Halictidae (*Lasioglossum,*  *Nomia*) | Pollinators |
| Hymenoptera | - Bethylidae, Crabronidae, Formicidae *(Anoplolepis, Anaplolepis*  *gracilipes, Crematogaster, Monomorium, Oecophylla*,  *Paratrechina, Pheidole, Polyrhachis, Tapinoma, Technomyrmex*), Mutillidae, Pompilidae, Scoliidae, Sphecidae, Tiphiidae, Vespidae | Predators |
| Lepidoptera | - Hesperiidae and Others | Pollinators |
