# Supplementary material for: Tree islands enhance biodiversity and functioning in oil palm landscapes
Source: Nature. 2023 May 24;618(7964):316–21. doi: 10.1038/s41586-023-06086-5 (PMC10247383; doi:10.1038/s41586-023-06086-5)
Supplement: Supplementary file 2 — Reporting Summary [file 41586_2023_6086_MOESM2_ESM.pdf]

nature portfolio

Corresponding author(s): Delphine Clara Zemp

Last updated by author(s): May 04, 2023

Reporting Summary

Nature Portfolio wishes to improve the reproducibility of the work that we publish. This form provides structure for consistency and transparency in reporting. For further information on Nature Portfolio policies, see our Editorial Policies and the Editorial Policy Checklist.

Statistics

For all statistical analyses, confirm that the following items are present in the figure legend, table legend, main text, or Methods section.

n/a	Confirmed	
☐	☒	The exact sample size (*n*) for each experimental group/condition, given as a discrete number and unit of measurement
☐	☒	A statement on whether measurements were taken from distinct samples or whether the same sample was measured repeatedly
☐	☒	The statistical test(s) used AND whether they are one- or two-sided<br>*Only common tests should be described solely by name; describe more complex techniques in the Methods section.*
☐	☒	A description of all covariates tested
☐	☒	A description of any assumptions or corrections, such as tests of normality and adjustment for multiple comparisons
☐	☒	A full description of the statistical parameters including central tendency (e.g. means) or other basic estimates (e.g. regression coefficient) AND variation (e.g. standard deviation) or associated estimates of uncertainty (e.g. confidence intervals)
☐	☒	For null hypothesis testing, the test statistic (e.g. *F*, *t*, *r*) with confidence intervals, effect sizes, degrees of freedom and *P* value noted<br>*Give P values as exact values whenever suitable.*
☐	☒	For Bayesian analysis, information on the choice of priors and Markov chain Monte Carlo settings
☐	☒	For hierarchical and complex designs, identification of the appropriate level for tests and full reporting of outcomes
☐	☒	Estimates of effect sizes (e.g. Cohen's *d*, Pearson's *r*), indicating how they were calculated

Our web collection on statistics for biologists contains articles on many of the points above.

Software and code

Policy information about availability of computer code

Data collection	The data were processed and analyzed in R version 1.2.1335
Data analysis	Data and R code are available at https://doi.org/10.6084/m9.figshare.22320490

For manuscripts utilizing custom algorithms or software that are central to the research but not yet described in published literature, software must be made available to editors and reviewers. We strongly encourage code deposition in a community repository (e.g. GitHub). See the Nature Portfolio guidelines for submitting code & software for further information.

Data

Policy information about availability of data

All manuscripts must include a data availability statement. This statement should provide the following information, where applicable:

- Accession codes, unique identifiers, or web links for publicly available datasets
- A description of any restrictions on data availability
- For clinical datasets or third party data, please ensure that the statement adheres to our policy

The raw data are available at https://data.goettingen-research-online.de/dataverse/crc990, with the specific link for each dataset provided in the Supplementary Tables 1 - 3. The processed data is available at https://doi.org/10.6084/m9.figshare.22320490.
Seed DNA sequences are available in NCBI Genbank under the accession numbers HYPERLINK "https://www.ncbi.nlm.nih.gov/nuccore/OM811991.1/" OM811991- HYPERLINK "https://www.ncbi.nlm.nih.gov/nuccore/OM812021" OM812021, HYPERLINK "https://www.ncbi.nlm.nih.gov/nuccore/OM837673" OM837673- HYPERLINK "https://www.ncbi.nlm.nih.gov/nuccore/OM837724" OM837724, and HYPERLINK "https://www.ncbi.nlm.nih.gov/nuccore/OM935782" OM935782- HYPERLINK "https://www.ncbi.nlm.nih.gov/nuccore/OM935815" OM935815. Sequencing data of the soil fungal community were deposited in the NCBI Sequence Read Archive (SRA) under Bioproject accession number PRJNA659225. The public UNITE database (https://unite.ut.ee/) v7.2 on fungal ITS sequences was used as a reference of taxonomic classification. Sequence data of the bacterial communities were deposited in the NCBI SRA under Bioproject accession number PRJNA841353. Sequence identification was performed by mapping all curated sequences against the SILVA database version 132 (https://www.arb-silva.de/).

Human research participants

Policy information about studies involving human research participants and Sex and Gender in Research.

Reporting on sex and gender: N / A

Population characteristics: N / A

Recruitment: N / A

Ethics oversight: N/A

Note that full information on the approval of the study protocol must also be provided in the manuscript.

Field-specific reporting

Please select the one below that is the best fit for your research. If you are not sure, read the appropriate sections before making your selection.

☐ Life sciences ☐ Behavioural & social sciences ☒ Ecological, evolutionary & environmental sciences

For a reference copy of the document with all sections, see nature.com/documents/nr-reporting-summary-flat.pdf

Ecological, evolutionary & environmental sciences study design

All studies must disclose on these points even when the disclosure is negative.

Study description: Our study was conducted in EFForTS-BEE, the Biodiversity Enrichment Experiment of the EFForTS project [Ecological and Socioeconomic Functions of Tropical Lowland Rainforest Transformation Systems (Sumatra, Indonesia)]. EFForTS-BEE is part of the global network of tree diversity experiments TreeDivNet (https://treedivnet.ugent.be/). The study region is characterized by a humid tropical climate with a mean temperature of 26.7 ± 0.2°C and an annual rainfall of 2,235 ± 381 mm and the dominant soil type is loamy Acrisol. In December 2013, 52 experimental plots (i.e. tree islands) were established in a conventional 140 ha oil palm plantation. Following a random partition design, we systematically varied plot area (25, 100, 400 and 1600 m2) and tree species diversity (0, 1, 2, 3 and 6 species). The six planted tree species (Archidendron jiringa (Jack) I.C.Nielsen. (Fabaceae), Parkia speciosa Hassk. (Fabaceae), Durio zibethinus L. (Malvaceae), Dyera polyphylla (Miq.) Steenis (Apocynaceae), Shorea leprosula Miq. (Dipterocarpaceae) and Peronema canescens Jack (Lamiaceae)) are native to the region and widely used for their fruits, timber or latex. Around 40% of the oil palms located inside the tree islands were felled, with the number of felled oil palms differed depending on the tree island area. The trees were planted between the felled and standing oil palms on a 2-m triangular grid. The tree islands were fenced, and the management comprised a total stop of fertilizer, herbicide and pesticide application after planting. After May 2016, manual weeding was restricted to 1-m circles around the planted trees when these were shorter than the surrounding grass layer, allowing for natural regeneration. In addition to the 52 tree islands, we established four control plots in the conventional oil palm plantation that were managed as usual, in the main text referred to as conventional monocultures. In total, the study comprised 56 plots. In each study plot larger than 25 m2, one subplot of 5 m x 5 m was established in a random location at a minimum distance of 1.5 m from the plot edge.

Research sample: Ecosystem functioning
We measured 20 variables related to seven categories of ecosystem functioning including Productivity: (0) tree growth (basal area increment of the planted trees in m2/ha/year) that was further excluded from the analysis - see method on multifunctionality, (1) Oil palm yield (per island oil palm yield changes in kg of fresh fruit bunches / island), (2) aboveground biomass (biomass stored in the aerial parts of the planted trees and the oil palms, in t/ha), Resistance to invasion: (3) native seeds (total number of arriving native seeds / m2); (4) resistance to invasive plants (100 – observed cover of Clidemia hirta, in %); Pollination: (5) pollinators (number of sampled individuals), (6) pollination rate (fraction of flowers on phytometer plants that are pollinated, %), Soil quality: (7) soil P (phosphorous content, %), (8) 1 / soil C:N (that is the molar ratio of soil C content to soil N content), (9) soil decompaction (inverse of soil bulk density in g/cm3); Predation and herbivory: (10) predatory invertebrates (total activity duration of insectivorous bats and birds, in seconds); (11) predatory arthropods (number of sampled individuals), (12) predatory soil fauna (energy flux, in J/hour), (13) herbivory (energy flux, in J/hour); Carbon & nutrient cycling: (14) Decomposers (energy flux, in J/hour); (15) litter decomposition (relative biomass loss of litter after 6 months in litterbags, %), (16) litter input (biomass of leaf litter falling in traps, g / m2), water and climate regulation: (17) evapotranspiration (canopy latent heat flux, in W/m2); (18) soil water infiltration capacity (saturated soil

hydraulic conductivity in cm/h), (19) micro-climate buffering (median daily amplitude of air temperature during one year, °C / day). A more detailed summary of the 20 ecosystem functioning variables is presented in Extended Data Table 1.
Biodiversity
We derived taxonomic diversity for soil bacteria and soil fungi, soil fauna, herbs, trees, seeds, pollen, understory arthropods, birds and bats. Most of the groups (arthropods, herbs, trees, birds, seeds) were sorted at the lowest possible taxonomic level (species or morphospecies). Pollen, soil fauna and bats were sorted to higher levels, mainly family, order and morphotypes, respectively. Soil bacteria and soil fungi were analyzed by DNA based marker gene sequencing as amplicons sequence variants (ASVs) or operational taxonomic units (OTU), respectively. Hereafter we refer to these different taxonomic units (species, family, order, morphotypes and OTU) as 'species' for simplicity.

Sampling strategy

In all the 56 study plots, multiple indicators related to biodiversity, ecosystem functioning, and structure were measured using standardized procedures and constant sampling areas at the level of the plot (i.e. tree island) or subplot (see Extended Data Tables 1-3). Only trees were sampled at unequal areas (i.e. all trees present in the plots were sampled) and were therefore standardized using rarefaction curves.

Data collection

In all the 56 study plots, multiple indicators related to biodiversity, ecosystem functioning, and structure were measured using standardized procedures and constant sampling areas at the level of the plot (i.e. tree island) or subplot (see Extended Data Tables 1-3).

Timing and spatial scale

We conducted an interdisciplinary field campaign from October 2016 to October 2018, i.e. 33 to 57 months after establishment of the experiment. At this early stage of the experiment, the tree islands already differed in their structural complexity and the planted trees reached up to 16 m height.

Data exclusions

As tree growth and leaf litter input were correlated and formed cluster, we excluded tree growth from the analysis. For leaf litter input, we then excluded outliers defined as plot-level values outside the range of 3 standard deviations around the median of the entire data (less than 5% of the litterweight data, total and per species).

Reproducibility

The Biodiversity Enrichment Experiment in Oil Palm Plantations (EFForTS-BEE) is unique but can be replicated to other landscapes. All data and code to reproduce the analyses are included at https://doi.org/10.6084/m9.figshare.22320490

Randomization

Each variable presented in the main text had one measurement per plot, such that randomization was not applicable.

Blinding

Each variable presented in the main text had one measurement per plot, such that blinding was not applicable.

Did the study involve field work? ☒ Yes ☐ No

Field work, collection and transport

Field conditions

The study region is characterized by a humid tropical climate with a mean temperature of 26.7 ± 0.2°C and an annual rainfall of 2,235 ± 381 mm and the dominant soil type is loamy Acrisol.

Location

The experiment was established on an oil- palm plantation of PT. Humusindo Makmur Sejati (01.95◦ S and 103.25◦ E, 47 ± 11 m a.s.l.) near Bungku village in the lowlands of Jambi province, Sumatra. The specific longitude and latitude for each plots are included in: https://doi.org/10.6084/m9.figshare.22320490

Access & import/export

Research permits were granted by the Indonesia Ministry of Research and Technology (Ristek or Ristekdikti) for all researchers involved in data collection for this study.
For soil fungi: The ITS2 sequences were deposited in the National Center for Biotechnology Information (NCBI) Sequence Read Archive (SRA) under bioproject accession number PRJNA659225.
For soil bacteria: The 16S rRNA gene and transcript sequences were deposited in the National Center for Biotechnology Information (NCBI) Sequence Read Archive (SRA) under bioproject accession number PRJNA841353

Disturbance

Major disturbance occured during the establishement of the experiment three to five years prior to the study presented here.

Reporting for specific materials, systems and methods

We require information from authors about some types of materials, experimental systems and methods used in many studies. Here, indicate whether each material, system or method listed is relevant to your study. If you are not sure if a list item applies to your research, read the appropriate section before selecting a response.

Materials & experimental systems

n/a	Involved in the study
☒	☐ Antibodies
☒	☐ Eukaryotic cell lines
☒	☐ Palaeontology and archaeology
☐	☒ Animals and other organisms
☒	☐ Clinical data
☒	☐ Dual use research of concern

Methods

n/a	Involved in the study
☒	☐ ChIP-seq
☒	☐ Flow cytometry
☒	☐ MRI-based neuroimaging

Animals and other research organisms

Policy information about studies involving animals; ARRIVE guidelines recommended for reporting animal research, and Sex and Gender in Research

Laboratory animals	No laboratory animals were used for this study.
Wild animals	Only invertebrate animals (arthropods and earthworms) were collected and killed using ethanol during the study. This was necessary to assess biomass/density and community composition. Soil animals were extracted from soil samples using a Kempson extractor under a heat gradient of 40-45 grad Celsius above and 15 grad Celsius below the samples. Animals were first collected in dimethyleneglycol - water solution (1:1) and thereafter transferred into 70-80% ethanol solution. Understorey arthropods were collected using pan traps that stayed for 45 hours in the field. Arthropods where directly killed in the bowl that is filled with water and one drop of scentless soap. Each pan trap was shaken off through a sieve so that the arthropods could be collected in the sieve and then stored in a test tube containing 70% ethanol. The animals were collected by Anton Potapov with the research permit: 349/SIP/FRP/ES/Dit.KI/X/2016 (Validity 5 October 2016 to 5 October 2017) and 54/EXT/SIP/FRP/ES/Dit.KI/IX/2017 (validity 04 October 2017 to 4 October 2018) and Isabelle Arimond with the research permit 370/SIP/FRP/ES/Dit. KI/X/2016 (Validity 20 October 2016 to 20 March 2017). Animal sex was not considered in the study.
Reporting on sex	
Field-collected samples	After each round of fieldwork, samples were taken to the lab for identifications. Collected soil samples were transported in the lab within 2-3 days for heat extraction. All extracted invertebrates were stored in 70-80% ethanol solution and sorted to high-rank taxa under dissecting microscope.
Ethics oversight	No ethical approval was required for this study as it complies with regulations of the Collaborative Research Center 990 (project ID 192626868 – SFB 990)

Note that full information on the approval of the study protocol must also be provided in the manuscript.
